# 1,4-Bis(4,5-dihydro-1*H*-imidazol-2-yl)benzene–terephthalic acid–water (1/1/4)

**DOI:** 10.1107/S1600536809040616

**Published:** 2009-10-10

**Authors:** Shao-Ming Shang, Su-Yun Li, Chun-Xia Ren, Xin Wang, Lu-De Lu

**Affiliations:** a School of Chemical and Material Engineering, Nanjing University of Science and Technology, 200 Xiaolingwei Road, Nanjing, Jiangsu Province 210094, People’s Republic of China; b Department of Public Education, Jiangxi Vocational & Technical College of Electricity, 8 Mailu Road, Nanchang, Jiangxi Province 330032, People’s Republic of China; c School of Chemical and Material Engineering, Jiangnan University, 1800 Lihu Road, Wuxi, Jiangsu Province 214122, People’s Republic of China

## Abstract

The asymmetric unit of the title compound, C_12_H_14_N_4_·C_8_H_6_O_4_·4H_2_O, consists of one half of the 1,4-bis­(4,5-dihydro-1*H-*imidazol-2-yl)benzene (bib) mol­ecule, one half of the terephthalic acid (TA) mol­ecule and two water mol­ecules. Both the bib and the TA mol­ecules reside on crystallographic inversion centers, which coincide with the centroids of the respective benzene rings. The bib and the TA, together with the water mol­ecules, are linked through inter­molecular O—H⋯O, O—H⋯N and N—H⋯O hydrogen bonds, forming a three-dimensional network of stacked layers. Weak inter­molecular C—H⋯O contacts support the stability of the crystal structure.

## Related literature

For general background, see: Jeffrey (1997 ▶). For hydrogen bonding in mol­ecular complexes of disubstituted biphenyls, see: Thaimattam *et al.* (1998 ▶). For the synthesis of the title compound, see: Ren *et al.* (2007 ▶). For related structures, see: Ren *et al.* (2007 ▶, 2009 ▶ and literature cited therein); Shang *et al.* (2009 ▶). For experimental refinement details, see: Nardelli, (1999 ▶).
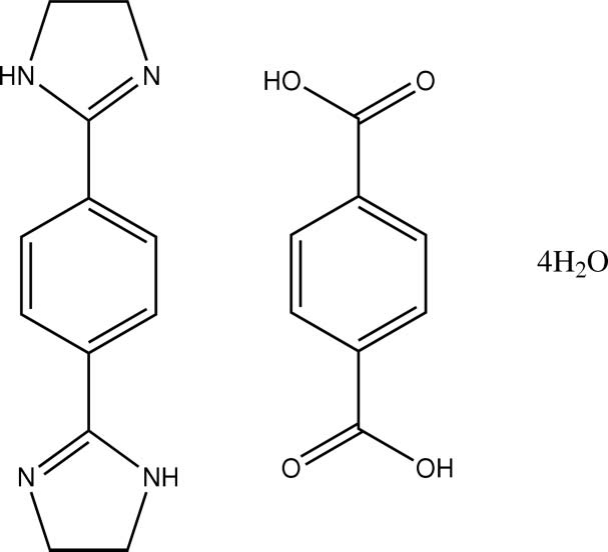


## Experimental

### 

#### Crystal data

C_12_H_14_N_4_·C_8_H_6_O_4_·4H_2_O
*M* *_r_* = 452.46
Monoclinic, 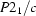

*a* = 7.9929 (13) Å
*b* = 16.847 (3) Å
*c* = 7.9615 (12) Å
β = 94.899 (3)°
*V* = 1068.2 (3) Å^3^
*Z* = 2
Mo *K*α radiation
μ = 0.11 mm^−1^
*T* = 273 K
0.50 × 0.35 × 0.30 mm

#### Data collection

Bruker SMART CCD area-detector diffractometer
Absorption correction: multi-scan (*SADABS*; Bruker, 1998 ▶) *T*_min_ = 0.947, *T*_max_ = 0.968
5588 measured reflections
2300 independent reflections
832 reflections with *I* > 2σ(*I*)
*R*_int_ = 0.075

#### Refinement

*R*[*F* ^2^ > 2σ(*F* ^2^)] = 0.090
*wR*(*F* ^2^) = 0.257
*S* = 0.79
2300 reflections
158 parameters
6 restraints
H atoms treated by a mixture of independent and constrained refinement
Δρ_max_ = 0.71 e Å^−3^
Δρ_min_ = −0.43 e Å^−3^

### 

Data collection: *SMART* (Bruker, 1998 ▶); cell refinement: *SAINT-Plus* (Bruker, 1998 ▶); data reduction: *SAINT-Plus*; program(s) used to solve structure: *SHELXS97* (Sheldrick, 2008 ▶); program(s) used to refine structure: *SHELXL97* (Sheldrick, 2008 ▶); molecular graphics: *SHELXTL* (Sheldrick, 2008 ▶) and *PLATON* (Spek, 2009 ▶); software used to prepare material for publication: *SHELXL97*.

## Supplementary Material

Crystal structure: contains datablocks global, I. DOI: 10.1107/S1600536809040616/si2204sup1.cif

Structure factors: contains datablocks I. DOI: 10.1107/S1600536809040616/si2204Isup2.hkl

Additional supplementary materials:  crystallographic information; 3D view; checkCIF report

## Figures and Tables

**Table 1:** Hydrogen-bond geometry (Å, °)

*D*—H⋯*A*	*D*—H	H⋯*A*	*D*⋯*A*	*D*—H⋯*A*
O2*W*—H2*WB*⋯O1^i^	0.85 (4)	2.37 (6)	2.904 (5)	121 (7)
O2*W*—H2*WA*⋯O1^ii^	0.85	1.96	2.815 (5)	180
O1*W*—H1*WB*⋯O2*W*^iii^	0.85 (4)	2.08 (4)	2.926 (5)	172 (5)
O1*W*—H1*WA*⋯O2^iv^	0.86 (3)	1.851 (15)	2.707 (5)	172 (3)
O2—H2*D*⋯N1	0.82	2.13	2.934 (4)	165
N2—H2*C*⋯O1*W*^v^	0.86	1.98	2.828 (4)	167
C5—H5⋯O1	0.93	2.50	3.407 (4)	165
C6—H6⋯O1*W*^v^	0.93	2.47	3.380 (6)	166

Symmetry codes: (i) 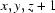; (ii) 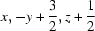; (iii) 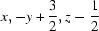; (iv) 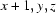; (v) 
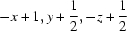
.

**Fig. 1.**
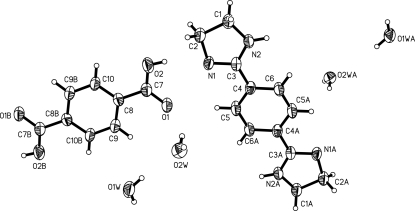
The molecular structure of the title molecule, with the atom-numbering scheme. Symmetry-related atoms shown labelled A and B. [symmetry codes A: (-x + 1, -y + 2, -z); B: (-x + 1, -y + 1, -z).

**Fig. 2.**
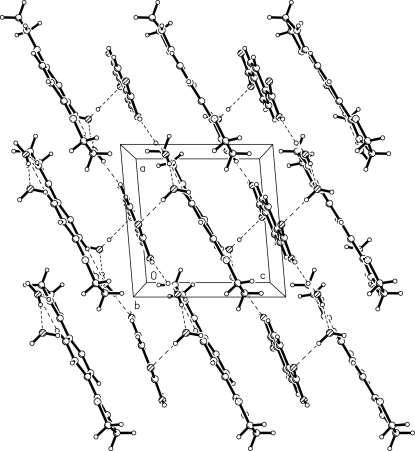
A partial packing diagram for the title compound. Hydrogen bonds are shown as dashed lines.

## supplementary crystallographic information

### Comment

Attention has recently focused on the use of supramolecular interactions such as hydrogen bonding and π–π interactions, in addition to coordinate bonds, in the controlled assembly of supramolecular architectures (Jeffrey, 1997). Hydrogen bonds often play a dominant role in crystal engineering because of their combine strength with directionality (Thaimattam, et al., 1998). On the other hand, supramolecular systems sustained by soft connections, such as hydrogen bonds, are comparatively more flexible and sensitive to the chemical environment. We described previously a number of such complexes, including the imidazole ligand, and have concluded that hydrogen bonding involving this group influences the geometry around the metal atom and the crystallization mechanism [Ren, et al. (2007, 2009 and literature cited therein); Shang et al. (2009)]. We describe herewith the synthesis and crystal structure of the title compound, (Fig. 1), namely bib.TA.4H_2_O (I) (bib = 1,4-bis(4,5-dihydro-1H-imidazol-2-yl)benzene, TA = terephthalate), which exhibits a three-dimensional hydrogen-bonded molecular architecture.

The crystal lattice contains two bib, two TA and eight lattice water molecules in the solid. The bib and TA in a trans, trans configuration are in a face-to-face orientation and the dihedral angle between acid TA and base bib components is 9.5°. And the bib and TA ligands are joined together by two water molecules through hydrogen bonds between the carboxy oxygen atom in TA and the nitrogen atom of –C=N– in bib to give a macrocycle O1W–H1WB···O2W, O2W—H2WB···O1, O1W—H1WA···O2, N2—H2C···O1W and O2–H2D···N1 with the hydrogen bond geometry given in Table 1, and a face-to-face intracyclic \<i>p-\<i>p interaction at 3.69 Å (Fig. 1). Each bib group also features another macrocycles, resulting in 1-D chains running along the a axis. As illustrated in Fig. 2, the adjacent TA ligands are furthermore linked in the antiparallel alignment with offset along the ab plane by hydrogen bonds between the water molecules and the oxygen of TA groups (O2W—H2WA···O1, O1W—H1WA···O2, O2W—H2WB···O1, and O1W—H1WB···O2W (see Table 1). These ab planes are packed and stabilized by the hydrogen bonds between the lattice water and oxygen atom of TA ligands (O2w—H2wa···O1 = 2.82 Å) into a 3-D structure. Weak intermolecular C—H···O contacts contribute to the stability of the layered structure (Table 1).

### Experimental

All the reagents and solvents employed were commercially available and used as received without further purification.

Syntheses of bib 1,4-Benzenedicarboxylic acid (2.31 g, 13.9 mmol), ethylenediamine (3.70 ml, 50 mmol), ethylenediamine dihydrochloride(6.64 g, 50 mmol) and toluene-*p*-sulfonic acid (0.208 g, 1.09 mmol) were added to the solvent of ethyleneglycol (20 ml), and the mixture solution was refluxed for 3 hr. About half of the ethylene glycol solvent was then slowly removed by distillation. The residue was dissolved in a mixture of water (40 ml) and concentrated HCl (11 *M*, 3 ml). The addition of 50% aqueous NaOH gave a yellow precipitate that was purified by recrystallization. The ligand bib was obtained in 83% based on 1,4-benzenedicarboxylic acid (*ca* 2.50 g). Found: C 66.98; H 6.92; N 26.08%. Calc. for C12H14N4: C 67.27; H 6.59; N 26.15%. Main IR bonds (KBr, cm^-1^): 3188*m*, 2936*m*, 2866*m*, 1606 s, 1532 s, 1466 s, 1345*m*, 1270 s, 1191w, 1080w, 981*m*, 907w, 767w, 687*m*.

Syntheses of bib.TA.4H_2_O (I) To a solution of bib (0.043 g, 0.2 mmol) in MeOH (15 ml), an aqueous solution (5 ml) of TA (0.034 g, 0.2 mmol) was added. The solution was allowed at room temperature in air for 48 hr by slow evaporation. Large colourless prismatic crystals of bib.TA.4H_2_O were obtained, which were collected by filtration, washed with water and dried in vacuum desiccator over silica gel (0.052 g, 56%). Found: C 53.02; H 6.20; N 12.31%. Calc. for C10H14N2O4: C 53.09; H 6.24; N 12.38%. Main IR bonds (KBr,cm^-1^): 3351*m*, 3142*m*, 2985*m*, 1626 s, 1601 s, 1582 s, 1516w, 1366 s, 1351 s, 1281*m*, 1040w, 864w, 822*m*, 752w, 689*m*.

### Refinement

All H atoms attached to C atoms and N atom were fixed geometrically and treated as riding with C—H = 0.93 Å (aromatic) or 0.97 Å (methylene) and N—H = 0.86 Å with *U*_iso_(H) = 1.2*U*_eq_(C or N). The positions of H atoms for water molecules were calculated (Nardelli, 1999) and included in the subsequent refinement as riding with *U*_iso_(H) = 1.5*U*_eq_(O).

### Crystal data

**Table:**

C_12_H_14_N_4_·C_8_H_6_O_4_·4H_2_O	*F*(000) = 480
*M**_r_* = 452.46	*D*_x_ = 1.407 Mg m^−^^3^
Monoclinic, *P*2_1_/*c*	Mo *K*α radiation, λ = 0.71073 Å
*a* = 7.9929 (13) Å	Cell parameters from 978 reflections
*b* = 16.847 (3) Å	θ = 2.4–22.2°
*c* = 7.9615 (12) Å	µ = 0.11 mm^−^^1^
β = 94.899 (3)°	*T* = 273 K
*V* = 1068.2 (3) Å^3^	Block, colorless
*Z* = 2	0.50 × 0.35 × 0.30 mm

### Data collection

**Table:**

Bruker SMART CCD area-detector diffractometer	2300 independent reflections
Radiation source: fine-focus sealed tube	832 reflections with *I* > 2σ(*I*)
graphite	*R*_int_ = 0.075
φ and ω scans	θ_max_ = 27.0°, θ_min_ = 2.4°
Absorption correction: multi-scan (*SADABS*; Bruker, 1998)	*h* = −10→10
*T*_min_ = 0.947, *T*_max_ = 0.968	*k* = −21→21
5588 measured reflections	*l* = −5→10

### Refinement

**Table:**

Refinement on *F*^2^	Secondary atom site location: difference Fourier map
Least-squares matrix: full	Hydrogen site location: inferred from neighbouring sites
*R*[*F*^2^ > 2σ(*F*^2^)] = 0.090	H atoms treated by a mixture of independent and constrained refinement
*wR*(*F*^2^) = 0.257	*w* = 1/[σ^2^(*F*_o_^2^) + (0.1628*P*)^2^] where *P* = (*F*_o_^2^ + 2*F*_c_^2^)/3
*S* = 0.79	(Δ/σ)_max_ < 0.001
2300 reflections	Δρ_max_ = 0.71 e Å^−^^3^
158 parameters	Δρ_min_ = −0.43 e Å^−^^3^
6 restraints	Extinction correction: *SHELXL97* (Sheldrick, 2008), Fc^*^=kFc[1+0.001xFc^2^λ^3^/sin(2θ)]^-1/4^
Primary atom site location: structure-invariant direct methods	Extinction coefficient: 0.12 (2)

### Special details

**Table:**

Geometry. All e.s.d.'s (except the e.s.d. in the dihedral angle between two l.s. planes) are estimated using the full covariance matrix. The cell e.s.d.'s are taken into account individually in the estimation of e.s.d.'s in distances, angles and torsion angles; correlations between e.s.d.'s in cell parameters are only used when they are defined by crystal symmetry. An approximate (isotropic) treatment of cell e.s.d.'s is used for estimating e.s.d.'s involving l.s. planes.
Refinement. Refinement of *F*^2^ against ALL reflections. The weighted *R*-factor *wR* and goodness of fit *S* are based on *F*^2^, conventional *R*-factors *R* are based on *F*, with *F* set to zero for negative *F*^2^. The threshold expression of *F*^2^ > σ(*F*^2^) is used only for calculating *R*-factors(gt) *etc*. and is not relevant to the choice of reflections for refinement. *R*-factors based on *F*^2^ are statistically about twice as large as those based on *F*, and *R*- factors based on ALL data will be even larger.

### Fractional atomic coordinates and isotropic or equivalent isotropic displacement parameters (Å^2^)

**Table:**

	*x*	*y*	*z*	*U*_iso_*/*U*_eq_	
N1	0.2209 (4)	0.83842 (17)	0.1428 (4)	0.0622 (10)	
N2	0.0932 (4)	0.95122 (17)	0.1837 (4)	0.0621 (10)	
H2C	0.0744	1.0015	0.1797	0.074*	
C1	−0.0182 (6)	0.8931 (2)	0.2516 (6)	0.0704 (13)	
H1A	−0.1318	0.8981	0.1991	0.085*	
H1B	−0.0199	0.8989	0.3726	0.085*	
C2	0.0597 (6)	0.8138 (2)	0.2069 (6)	0.0692 (13)	
H2A	0.0785	0.7800	0.3053	0.083*	
H2B	−0.0109	0.7860	0.1208	0.083*	
C3	0.2264 (5)	0.9167 (2)	0.1292 (4)	0.0558 (11)	
C4	0.3678 (5)	0.9588 (2)	0.0625 (4)	0.0541 (11)	
C5	0.4927 (6)	0.9183 (2)	−0.0102 (5)	0.0684 (13)	
H5	0.4886	0.8632	−0.0177	0.082*	
O1	0.4731 (4)	0.71811 (16)	0.0453 (4)	0.0702 (9)	
O2	0.2324 (5)	0.66542 (18)	0.1033 (4)	0.0915 (11)	
H2D	0.2110	0.7124	0.1183	0.137*	
C6	0.3758 (6)	1.0410 (2)	0.0723 (5)	0.0659 (12)	
H6	0.2921	1.0689	0.1212	0.079*	
C7	0.3793 (7)	0.6590 (2)	0.0620 (5)	0.0649 (12)	
C8	0.4386 (5)	0.5771 (2)	0.0299 (5)	0.0570 (11)	
C9	0.6008 (6)	0.5648 (2)	−0.0226 (5)	0.0647 (12)	
H9	0.6694	0.6082	−0.0391	0.078*	
C10	0.3416 (6)	0.5107 (2)	0.0509 (5)	0.0644 (12)	
H10	0.2339	0.5170	0.0849	0.077*	
O1W	0.9737 (5)	0.61448 (19)	0.2736 (5)	0.0901 (12)	
H1WA	1.053 (4)	0.627 (2)	0.212 (5)	0.11 (2)*	
H1WB	0.904 (5)	0.6509 (19)	0.296 (6)	0.10 (2)*	
O2W	0.7204 (4)	0.76340 (18)	0.8161 (5)	0.0821 (11)	
H2WA	0.6459	0.7691	0.7341	0.098*	
H2WB	0.700 (6)	0.776 (7)	0.916 (3)	0.31 (7)*	

### Atomic displacement parameters (Å^2^)

**Table:**

	*U*^11^	*U*^22^	*U*^33^	*U*^12^	*U*^13^	*U*^23^
N1	0.076 (2)	0.0398 (17)	0.073 (2)	−0.0058 (16)	0.0184 (18)	0.0054 (15)
N2	0.074 (2)	0.0401 (18)	0.074 (2)	0.0037 (17)	0.0201 (19)	0.0008 (15)
C1	0.082 (3)	0.050 (2)	0.082 (3)	−0.003 (2)	0.021 (2)	0.002 (2)
C2	0.087 (3)	0.047 (2)	0.076 (3)	−0.002 (2)	0.022 (2)	0.0008 (19)
C3	0.078 (3)	0.040 (2)	0.050 (2)	0.000 (2)	0.007 (2)	0.0006 (16)
C4	0.072 (3)	0.043 (2)	0.048 (2)	−0.0032 (19)	0.015 (2)	0.0032 (16)
C5	0.094 (3)	0.039 (2)	0.077 (3)	0.003 (2)	0.033 (3)	−0.003 (2)
O1	0.089 (2)	0.0440 (15)	0.080 (2)	0.0001 (15)	0.0227 (16)	−0.0008 (13)
O2	0.099 (3)	0.0495 (17)	0.132 (3)	0.0123 (16)	0.046 (2)	−0.0028 (16)
C6	0.079 (3)	0.042 (2)	0.081 (3)	0.004 (2)	0.029 (2)	0.0007 (19)
C7	0.080 (3)	0.050 (2)	0.067 (3)	0.003 (2)	0.018 (2)	0.0043 (19)
C8	0.069 (3)	0.047 (2)	0.056 (2)	0.0062 (19)	0.012 (2)	0.0006 (17)
C9	0.082 (3)	0.042 (2)	0.071 (3)	−0.007 (2)	0.011 (2)	−0.0018 (19)
C10	0.080 (3)	0.040 (2)	0.075 (3)	−0.0062 (19)	0.020 (2)	−0.0006 (18)
O1W	0.100 (3)	0.0528 (19)	0.123 (3)	−0.0071 (19)	0.038 (2)	0.0076 (17)
O2W	0.076 (2)	0.077 (2)	0.094 (3)	0.0043 (16)	0.0140 (17)	0.0025 (18)

### Geometric parameters (Å, °)

**Table:**

N1—C3	1.324 (5)	O2—C7	1.251 (5)
N1—C2	1.485 (5)	O2—H2D	0.8200
N2—C3	1.319 (5)	C6—C5^i^	1.381 (5)
N2—C1	1.458 (5)	C6—H6	0.9300
N2—H2C	0.8600	C7—C8	1.487 (6)
C1—C2	1.528 (5)	C8—C10	1.379 (5)
C1—H1A	0.9700	C8—C9	1.412 (6)
C1—H1B	0.9700	C9—C10^ii^	1.377 (5)
C2—H2A	0.9700	C9—H9	0.9300
C2—H2B	0.9700	C10—C9^ii^	1.377 (5)
C3—C4	1.472 (6)	C10—H10	0.9300
C4—C5	1.377 (5)	O1W—H1WA	0.86 (3)
C4—C6	1.389 (5)	O1W—H1WB	0.86 (4)
C5—C6^i^	1.381 (5)	O2W—H2WA	0.85
C5—H5	0.9299	O2W—H2WB	0.85 (4)
O1—C7	1.260 (5)		
			
C3—N1—C2	110.0 (3)	C4—C5—C6^i^	120.4 (3)
C3—N2—C1	111.2 (3)	C4—C5—H5	119.9
C3—N2—H2C	124.4	C6^i^—C5—H5	119.7
C1—N2—H2C	124.4	C7—O2—H2D	109.5
N2—C1—C2	103.1 (3)	C5^i^—C6—C4	120.5 (4)
N2—C1—H1A	111.1	C5^i^—C6—H6	119.7
C2—C1—H1A	111.1	C4—C6—H6	119.8
N2—C1—H1B	111.1	O2—C7—O1	122.7 (4)
C2—C1—H1B	111.1	O2—C7—C8	116.4 (4)
H1A—C1—H1B	109.1	O1—C7—C8	120.9 (4)
N1—C2—C1	102.7 (3)	C10—C8—C9	117.1 (3)
N1—C2—H2A	111.2	C10—C8—C7	122.8 (4)
C1—C2—H2A	111.2	C9—C8—C7	120.1 (4)
N1—C2—H2B	111.2	C10^ii^—C9—C8	120.9 (4)
C1—C2—H2B	111.2	C10^ii^—C9—H9	119.6
H2A—C2—H2B	109.1	C8—C9—H9	119.5
N2—C3—N1	112.3 (4)	C9^ii^—C10—C8	122.0 (4)
N2—C3—C4	124.9 (3)	C9^ii^—C10—H10	118.8
N1—C3—C4	122.8 (4)	C8—C10—H10	119.1
C5—C4—C6	119.1 (4)	H1WA—O1W—H1WB	118 (2)
C5—C4—C3	121.3 (3)	H2WA—O2W—H2WB	120.8
C6—C4—C3	119.6 (4)		
			
C3—N2—C1—C2	−7.5 (4)	C3—C4—C5—C6^i^	179.9 (4)
C3—N1—C2—C1	−7.6 (4)	C5—C4—C6—C5^i^	−0.1 (7)
N2—C1—C2—N1	8.6 (4)	C3—C4—C6—C5^i^	−179.9 (4)
C1—N2—C3—N1	2.9 (5)	O2—C7—C8—C10	−2.8 (6)
C1—N2—C3—C4	−176.3 (4)	O1—C7—C8—C10	178.3 (4)
C2—N1—C3—N2	3.3 (5)	O2—C7—C8—C9	177.9 (4)
C2—N1—C3—C4	−177.5 (3)	O1—C7—C8—C9	−1.0 (6)
N2—C3—C4—C5	−172.2 (4)	C10—C8—C9—C10^ii^	−0.1 (6)
N1—C3—C4—C5	8.6 (6)	C7—C8—C9—C10^ii^	179.2 (4)
N2—C3—C4—C6	7.6 (6)	C9—C8—C10—C9^ii^	0.1 (6)
N1—C3—C4—C6	−171.6 (4)	C7—C8—C10—C9^ii^	−179.2 (4)
C6—C4—C5—C6^i^	0.1 (7)		

Symmetry codes: (i) −*x*+1, −*y*+2, −*z*; (ii) −*x*+1, −*y*+1, −*z*.

### Hydrogen-bond geometry (Å, °)

**Table:**

*D*—H···*A*	*D*—H	H···*A*	*D*···*A*	*D*—H···*A*
O2W—H2WB···O1^iii^	0.85 (4)	2.37 (6)	2.904 (5)	121 (7)
O2W—H2WA···O1^iv^	0.85	1.96	2.815 (5)	180
O1W—H1WB···O2W^v^	0.85 (4)	2.08 (4)	2.926 (5)	172 (5)
O1W—H1WA···O2^vi^	0.86 (3)	1.85 (2)	2.707 (5)	172 (3)
O2—H2D···N1	0.82	2.13	2.934 (4)	165
N2—H2C···O1W^vii^	0.86	1.98	2.828 (4)	167
C5—H5···O1	0.93	2.50	3.407 (4)	165
C6—H6···O1W^vii^	0.93	2.47	3.380 (6)	166

Symmetry codes: (iii) *x*, *y*, *z*+1; (iv) *x*, −*y*+3/2, *z*+1/2; (v) *x*, −*y*+3/2, *z*−1/2; (vi) *x*+1, *y*, *z*; (vii) −*x*+1, *y*+1/2, −*z*+1/2.
